# Fabrication of Microstructured Surface Topologies for the Promotion of Marine Bacteria Biofilm

**DOI:** 10.3390/mi12080926

**Published:** 2021-08-03

**Authors:** Ariadni Droumpali, Jörg Hübner, Lone Gram, Rafael Taboryski

**Affiliations:** 1 National Centre for Nano Fabrication and Characterization, Technical University of Denmark, 2800 Kongens Lyngby, Denmark; adro@dtu.dk (A.D.); jhub@dtu.dk (J.H.); 2 Department of Biotechnology and Biomedicine, Technical University of Denmark, 2800 Kongens Lyngby, Denmark; gram@bio.dtu.dk

**Keywords:** structured surfaces, silicon surfaces, microfabrication, bacterial biofilm, microbial adhesion

## Abstract

Several marine bacteria of the *Roseobacter* group can inhibit other microorganisms and are especially antagonistic when growing in biofilms. This aptitude to naturally compete with other bacteria can reduce the need for antibiotics in large-scale aquaculture units, provided that their culture can be promoted and controlled. Micropatterned surfaces may facilitate and promote the biofilm formation of species from the *Roseobacter* group, due to the increased contact between the cells and the surface material. Our research goal is to fabricate biofilm-optimal micropatterned surfaces and investigate the relevant length scales for surface topographies that can promote the growth and biofilm formation of the *Roseobacter* group of bacteria. In a preliminary study, silicon surfaces comprising arrays of pillars and pits with different periodicities, diameters, and depths were produced by UV lithography and deep reactive ion etching (DRIE) on polished silicon wafers. The resulting surface microscale topologies were characterized via optical profilometry and scanning electron microscopy (SEM). Screening of the bacterial biofilm on the patterned surfaces was performed using green fluorescent staining (SYBR green I) and confocal laser scanning microscopy (CLSM). Our results indicate that there is a correlation between the surface morphology and the spatial organization of the bacterial biofilm.

## 1. Introduction

Marine aquaculture industries are continuously expanding due to the increasing demand for fish production to feed the growing world population [1]. Infectious bacterial diseases on the rearing units of many fish species are common; therefore, the use of antibiotics is increasing [2,3]. Consequently, the risk of antibiotic resistance is higher, and new sustainable alternatives for disease control are essential [4,5].

Several studies have shown that marine bacteria of the *Roseobacter* group, such as the well-studied *Phaeobacter inhibens*, can inhibit other microorganisms, such as the fish pathogenic *Vibrionaceae*, by producing the antibacterial compound tropodithietic acid (TDA) [6,7,8,9]. Several of the probiotic bacteria are especially antagonistic when growing in biofilms [10,11].

One of the main challenges in using probiotic bacteria is how to optimally introduce the beneficial bacteria into the rearing units. Bentzon-Tilia et al. (2016) suggested the integration of the probiotic bacteria into synthetic bacterial communities of pre-established biofilms of biological aerated filters (BAFs) in recirculating aquaculture systems (RAS). Moreover, biofilms on synthetic communities could be released and allow the spread of probiotics throughout the system [12].

A better understanding of how a given surface can promote or inhibit biofilm attachment is crucial for potential applications. In this study, we focus on how to manipulate surface topography in order to enhance beneficial bacterial biofilms relevant to aquaculture systems. Our focus is on the enhancement of biofilm formation of the *Roseobacter* group, especially the TDA-producing *Phaeobacter* species. 

Several studies have shown that many factors can influence bacterial attachment on surfaces [13,14,15,16,17,18]. Most of these studies have focused on antibacterial properties and surface morphologies. Based on microscale topographies, micropatterned surfaces may promote biofilm formation due to increased contact between the cells and the surface material [19,20,21]. On the other hand, an emphasis on surface morphology and the promotion of biofilms is crucial. In this case, the aim is to identify how microcavities can be integrated in industrial applications for the promotion of beneficial bacterial biofilms.

In our work, we explore the attachment of *Phaeobacter inhibens* on micropatterned silicon surfaces in order to comprehend how a particular surface morphology can influence bacterial attachment to the surface. By applying UV lithography and DRIE, we investigate how microfabricated silicon surfaces with different morphologies, such as pits or pillars, aspect ratios, and length scales of honeycomb-patterned surfaces, can maximize the attachment of biofilm in comparison to planar reference surfaces [22,23]. 

## 2. Materials and Methods

### 2.1. Fabrication of Silicon Surfaces

Silicon surfaces with microcavities, comprising arrays of hexagonal pillars and pits with different periodicities, diameters, and depths, were originated by conventional UV lithography [24] and etched by deep reactive ion etching (DRIE) [25] on 100 mm diameter n-type <100> single-sided polished silicon wafers with a thickness of 525 ± 20 µm (Siegert Wafer GmbH).

A thin layer of 1.5 µm photoresist (AZ5214E) was applied to the 100 mm silicon wafers using a spin coater (Gamma 2M cluster, Süss MicroTec, Garching, Germany). The patterns were designed using CleWin5 software (WieWeb software, Hengelo, The Netherlands), and then the design was transferred to the photoresist using a Maskless h-line Aligner (Heidelberg Instruments MLA150 Maskless Aligner) by means of an 8W laser and a dose of 65 mJ/cm^2^, emitting at 405 nm. The samples were developed for 60 s in a TMAH-based solution (AZ 726 MIF—2.38% TMAH in water) to expose the pattern (Süss MicroTec Gamma 2M developer, s/n GAMMA-000233). Subsequently, the wafers were etched via a DRIE process (STS Pegasus DRIE) using a Bosch process to a target depth of 10 μm (Table S1) [25,26,27,28]. An oxygen plasma-based ashing process (Table S2) completely removed the remaining photoresist and etching residues from the Bosch process. Several identical wafers were fabricated. The fabrication steps are shown in Figure S1. The fabricated silicon wafers were cut into microscopy slide shapes of size 25 mm × 75 mm for easier handling during experimental tests with bacterial attachment and microscopy (Section 2.5 and Section 2.6).

#### Sizes of the Designed Honeycomb Patterns

The patterned surfaces were designed to have different dimensions and periodicities. Arrays of honeycomb pillars and pits were designed with trench widths and wall widths, respectively, of 1, 2.5, and 5 µm and side lengths a of 2.5, 5, and 10 µm. In Table S3, the designed dimensions of the honeycomb pillars are presented. In Table S4, the different designed dimensions of the honeycomb pits are shown. Planar surfaces, which are 2-dimensional flat surfaces, were used as controls for comparison with the designed patterns. The planar surfaces that were used as control surfaces originated from the same wafer as the micropatterned surfaces and thus underwent the same treatment during the fabrication process.

### 2.2. Characterization via Scanning Electron Microscopy (SEM)

SEM imaging (Zeiss Supra VP 40, Carl Zeiss AG, Jena, Germany) was used to acquire micrographs of the micropatterned surfaces. The microscope was connected to SmartSEM software (Carl Zeiss AG, Jena, Germany), used for imaging and analysis. The accelerating voltage was between 3 and 5 kV. The actual trench width (d) and side length (a) of the different structures were determined from SEM images. The height (h) was determined from SEM images acquired by tilting the stage at an angle of either 30° or 45°.

### 2.3. Characterization via Optical Profilometry

For depth measurements of the microstructures on the silicon masters, a confocal profilometer (S Neox 3D Optical Profiler, Terrassa, Spain) was used. The acquisition of the 3D profiles was performed using a basic confocal mode (with a 50×/0.80 objective). An average of 3 images and a speed factor of 1× were used for higher resolution. SensoMAP, an advanced analysis software product, was used for the analysis of the acquired 3D profiles [29]. The optical profiler was operated to measure the etch depth on the fabricated silicon wafers. 

### 2.4. Bacterial Strain and Culture Conditions

The *Phaeobacter inhibens* DSM 17395 strain, which belongs to the *Roseobacter* group, was used for the biofilm formation experiments. Bacterial stock cultures were stored at −80 °C in 20% (vol/vol) glycerol. Two to three days prior to use, stock cultures were streaked on Marine Agar plates (Marine Agar, Difco 2216) and incubated at 25 °C. Single colonies were used for the inoculation of each preculture. All bacterial precultures were grown in 20 mL of MB (Marine Broth, Difco 2216) at 25 °C for 24 h.

### 2.5. Cell Adhesion Experiments

Cell adhesion tests with *Phaeobacter inhibens* DSM 17395 were performed in closed batch systems under different growth conditions.

The first set of experiments was conducted by inoculating a volume from the undiluted precultures of *P. inhibens,* which was added to 250 mL mineral media [30] supplemented with 0.1% glucose to reach a starting concentration of 10^4^ CFU mL^−1^. The silicon microscopy slides, fabricated as described in Section 2.1, were sterilized by autoclaving at 121 °C for 15 min. Three sterilized slides were then placed, on a metal rack, on the beaker with 250 mL inoculum (Figure S2). The slides were incubated with bacteria at 25 °C for 24, 48, or 72 h, under constant stirring at 100 rpm. 

The second series of experiments was performed following the same bacteria inoculation procedure, but changing the following conditions: the stirring was increased to 700 rpm, and the incubation time was extended up to 192 h (8 days).

### 2.6. Microscopy Parameters, Image Acquisition, and Analysis

Microscopic observations of bacterial biofilms and microscopy slides were obtained using an inverted Leica TCS SP8 confocal laser scanning microscope (Leica Microsystems, Mannheim, Germany) equipped with an argon/krypton laser and detectors and filter sets for monitoring GFP (excitation: 488 nm, emission: 493–558 nm) for cell imaging. Images were obtained using an HC PL Apo CS2 63×/1.40 dry objective.

Bacterial biofilms were stained with SYBR^®^ Green PCR Master Mix (4309155; Applied Biosystems) using the following procedure: 2.5 µL of 10% SYBR green I was diluted in 97.5 µL of milliQ water; then, 12.5 µL of the diluted SYBR green was used to stain each slide. Each microscope slide was allowed to dry for 15 min in the dark before being washed with a 3% sea salt solution (3% sea salt (S9883; Sigma–Aldrich)) by dipping them in and taking them out 3 times. After washing, the slides were allowed to dry for 20 to 30 min in the dark. A mounting buffer (p-phenylene-diamine from Sigma-Aldrich, P600 in 43% glycerol), diluted to a final concentration of 0.1% in phosphate-buffered saline (PBS), was added to reduce the fading of the SYBR green staining. After adding the mounting buffer and before microscopy observations, a rectangular glass cover slide with a size of 50 × 24 mm and a thickness of 170 µm was placed on top of the samples.

Confocal microscopy images were acquired at z-intervals of 1 µm. As a control, three biological experiments on the silicon planar surfaces were performed, acquiring three images (technical replicates) at random positions to account for any heterogeneity within the biofilm. Stacked images were generated using Imaris software (Version 7.7.1, Bitplane AG, Zürich, Switzerland). The volume of biomass was calculated using the image-analysis COMSTAT version 2.1 software [31,32]. Data are expressed as the mean ± SD unless otherwise noted. Graphs were made and statistical analyses were conducted in Origin Pro 2019 (Version 9.6.0.172, OriginLab Corporation, Northampton, MA, USA).

## 3. Results

### 3.1. Fabricated Honeycomb Patterns

#### Measured Dimensions of the Patterned Surfaces

The dimensions and sizes of all the fabricated honeycomb patterns were measured by either SEM or optical profilometry as described in Section 2.2 and Section 2.3. In Figures S3 and S4, the measured values of the trench widths, side lengths, and heights are compared with the nominal values of both honeycomb pillars (Hs) and honeycomb pits (Rs).

**Figure 1:**
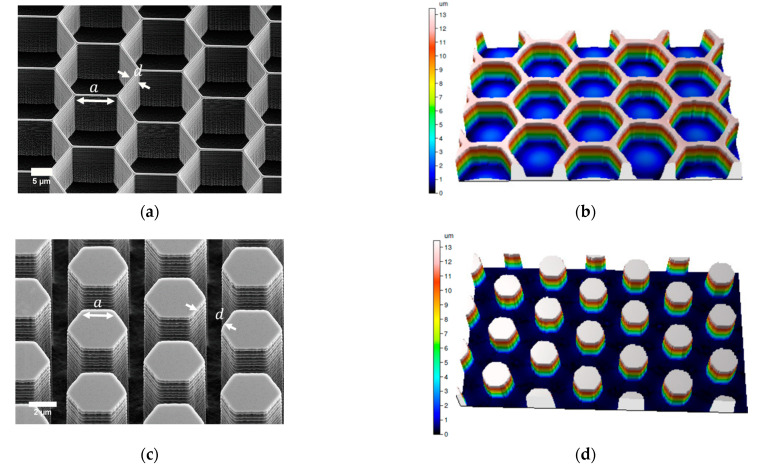
Scanning electron micrographs and matching 3D profiles, taken using an optical profiler (Sensofar PLu Neox 3D), of different microstructured honeycomb patterns after the silicon etch process. (**a**) SEM image of an array of honeycomb pits with side length a and wall width d; (**b**) Corresponding 3D profile of the honeycomb pits; (**c**) SEM image of an array of honeycomb pillars; (**d**) Corresponding 3D profile of the honeycomb pillars.

In Figure 1, the dimensions of two different patterns are described. In Figure 1a, a SEM micrograph of an array of honeycomb pits is shown. In Figure 1b, the corresponding 3D profile, taken using an optical profiler (Sensofar PLu Neox 3D), of the pattern is illustrated. The depth measured by Sensofar was ~12 µm and the side length a was around 10 µm, with wall width d ~ 2.5 µm. In Figure 1c, a SEM micrograph of an array of honeycomb pillars is presented. In Figure 1d, the corresponding 3D profile of the pattern is shown. The measured depth was ~13 µm and the side length a was ~2.5 µm, with wall width d ~2.5 µm.

### 3.2. Impact of the Fabricated Patterns on Bacterial Biofilm Attachment

To determine whether the patterned surfaces have an impact on bacterial attachment, a series of bacterial attachment experiments were conducted as described in Section 2.5. 

**Figure 2:**
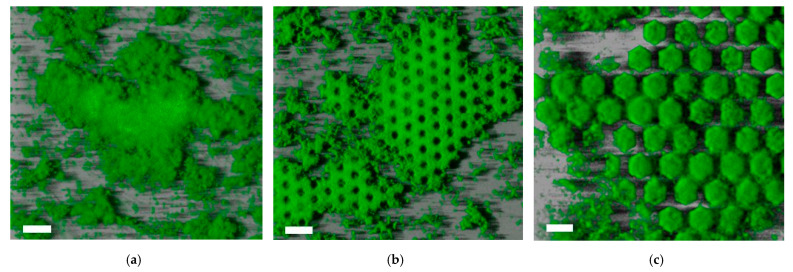
Confocal laser scanning micrographs showing bacterial biofilm of *P. inhibens* attached on the fabricated silicon surfaces. Biofilms were incubated with low stirring for 48 h at 25 °C: (**a**) Biofilm grown on a planar surface; (**b**) Biofilm grown on honeycomb pillars; (**c**) Biofilm grown on honeycomb pits. Scale bar is 20 µm.

Bacterial attachment on patterned surfaces with different polarities (pillars and pits) and different length scales was first examined as a function of time. Screening of the bacterial biofilm on the patterned surfaces was performed using green fluorescent staining (SYBR green I) and confocal laser scanning microscopy (CLSM). CLSM images were acquired after 24, 48, and 72 h of *P. inhibens* incubation at 25 °C and at 100 rpm. Confocal images of *P. inhibens* bacterial biofilm attachment on a planar surface and on patterned honeycomb pillars and honeycomb pits after 48 h are shown in Figure 2. 

**Figure 3:**
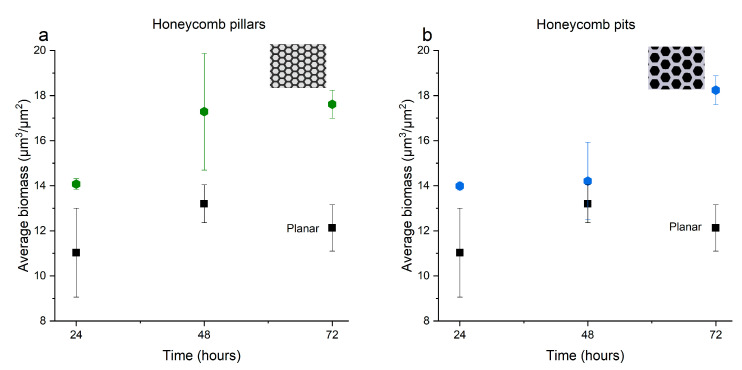
Composite graph illustrating the average bacterial biofilm biomass (µm^3^ µm^−2^) (bacterial volume/surface area) of *Phaeobacter inhibens* grown on the different surfaces, as a function of time: (**a**) Average biomass of bacterial biofilm grown on honeycomb pillars with a trench width of 5 µm, a side length of 10 µm, and a depth of 12 µm; (**b**) Average biomass of bacterial biofilm grown on honeycomb pits with a trench width of 5 µm, a side length of 10 µm, and a height of 13 µm. Data are depicted as mean + SD (*n* = 3).

A quantitative analysis of the confocal images was carried out to obtain the bacterial biomass (bacterial volume/surface area, cubic micrometers per square micrometer (µm^3^ µm^−2^)). The average bacterial biomass was quantified using three images (technical replicates) of each patterned surface, and the mean value with standard deviation is presented. As shown in Figure 3, the average bacterial biomass on planar surfaces was around 12 µm^3^ µm^−2^ after 3 days of growth, which was slightly lower than that after 48 h (13.2 µm^3^ µm^−2^). The patterned surface consisting of an array of pillars with parameters a = 10 µm and d = 5 µm showed greater growth both after 48 and after 72 h than did the planar reference (Figure 3a). The average biomass of the grown biofilm after 72 h was higher on honeycomb pits with parameters a = 10 µm and *d* = 5 µm than on the planar reference (Figure 3b).

**Figure 4:**
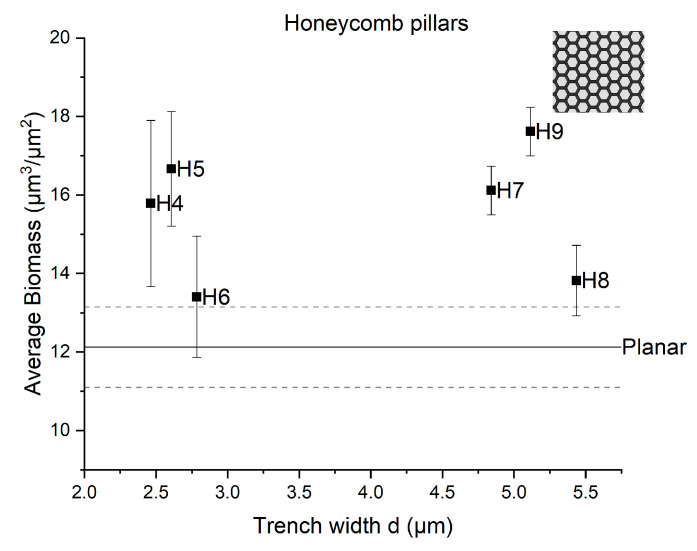
Average bacterial biofilm biomass (µm^3^ µm^−2^) (bacterial volume/surface area) of *Phaeobacter inhibens biofilm* on the honeycomb pillar surfaces, as a function of trench width d. Data are depicted as mean + SD (*n* = 3). The straight line serves as a baseline, as it indicates the average biomass on the planar surface, with the standard deviation for the planar surface shown with dashed lines.

In Figure 4, the average bacterial biofilm biomass of the grown biofilm on patterned surfaces with honeycomb pillars is shown as a function of the trench width d. The biofilm biomass on the planar flat surface was 12.13 ± 1.03 µm^3^/µm^2^. The highest biofilm biomass is shown for the honeycomb pits H9. It can be observed that the average biomass values of honeycomb pillars H4, H5, and H7 were also higher than the biomass value on the planar surface. However, for the surfaces H6 and H8, the average biomass values were similar to that for the planar surface. 

**Figure 5:**
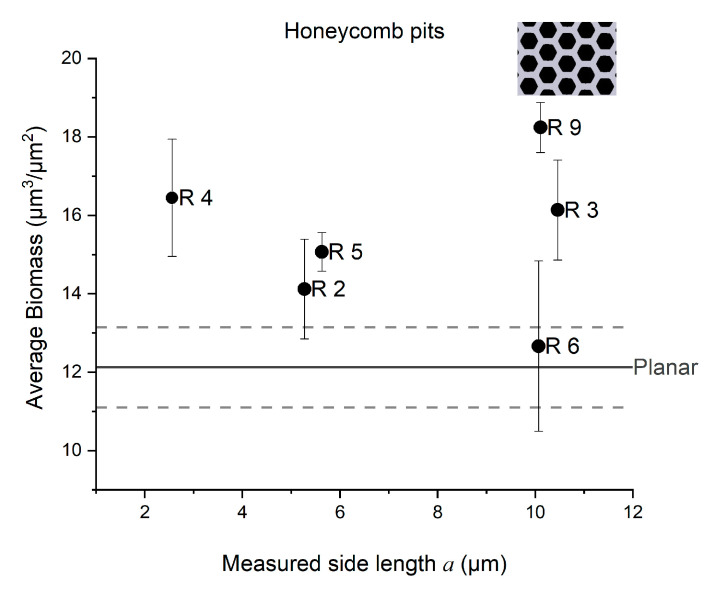
Average bacterial biofilm biomass (µm^3^ µm^−2^) (bacterial volume/surface area) of *Phaeobacter inhibens biofilm* on the different pit surfaces, as a function of side length a. The straight line serves as a baseline, as it indicates the average biomass on the planar surface, with the standard deviation for the planar surface shown with dashed lines. Data are depicted as mean + SD (*n* = 3).

The average biomass of the grown biofilm was higher on honeycomb pits R9 than on the planar surface (Figure 5). A higher biofilm biomass was also shown for the honeycomb pits R3, but not for R6. These results suggest that the trench width has an effect on the biofilm growth on pillar array surfaces, whereas the side length has an effect on biofilm grown on patterned surfaces with honeycomb pits.

## 4. Discussion

We tested whether a surface feature length scale could increase bacterial attachment by modifying the surface area. Studies comprising surfaces with micro- and nanostructures that influence bacterial adhesion have mainly focused on antibacterial properties and surface morphologies [13,14,15,17,33,34,35,36]. Here, we fabricated hexagonal micropatterned surfaces with different polarities and different length scales, such as side lengths, trench widths, and depths (Figure 1). The fabricated surfaces were based on a honeycomb pattern, which was chosen because it is a widely used, naturally occurring pattern [37] that has been previously studied for bacterial attachment [19,21].

The fabricated substrates were silicon surfaces, and the patterned designs were etched via a DRIE etching Bosch process (Section 2.1, Table S1). The etched surfaces after this process were not as smooth as a pristine planar flat surface. As shown in Figure 1a,b, after the dry etching, the pillars and sidewalls of the pits had a certain roughness due to periodic undulations, also known as scallops [38]. Surface roughness can prevent or promote the attachment of biofilm on fabricated surfaces [18,39]. The formation of scallops increases the surface area, so bacterial attachment may be enhanced. In our study, the effect of the produced scallops on the honeycomb pits may also have been beneficial.

By employing confocal microscopy to quantify the bacterial growth, we demonstrated that both honeycomb pillar and pit arrays resulted in higher biomass than the planar reference surface over time. Specifically, a pillar array surface with a= 10 µm and d= 5 µm showed greater growth, both after 48 and after 72 h (Figure 3a), compared to the flat surface. Also, in Figure 3b, we see that the average biomass on the honeycomb pits with side length a=10 µm and d= 5 µm was higher than that on the planar surface after 72 h. These results indicate that the bacterial biofilm volume corresponding to a surface (biomass) can be controlled by changing the trench width between honeycomb pillars and the side length of honeycomb pits. Scheuerman et al. (1998) studied bacterial attachment on microscale topographies and stated that bacterial attachment is independent of groove width [20]. Furthermore, their study suggested that only the motility of strains allows cells to accumulate on the bottom of grooves [20]. Our preliminary study is thus in disagreement with the findings of Scheuerman et al. (1998). In Figure 5, we can observe that different side lengths and cavities might also influence bacterial biofilm attachment on patterned surfaces. Our observations are supported by those reported in a recent review, which showed that there is an interaction of bacterial adhesion on patterned surfaces [15]. 

A limitation in this preliminary study is concerned with the experimental setup, which consisted of a closed system, without a continuous supply of nutrients, and the fixed orientation of the silicon slides. Growth media were added only at the beginning of the experiment, and this may have influenced biofilm maturing and aging, as overgrown biofilms were encountered in some cases (Figure S5). Furthermore, even though the patterned surfaces where randomized on the silicon microscope slides and the stirring was kept constant during the experiment; the orientation of the slides might have had an influence on the bacterial attachment (Figure S2). Overall, overcoming the mentioned limitations could lead to a better understanding of biofilm growth promotion by means of microfabricated patterns.

As previously mentioned, the material used in our pilot experiment was pure silicon. Another material with a different surface energy could very well result in enhanced biofilm attachment [40,41,42,43,44,45]. Finally, in this study, we used patterned surfaces with heights of around 13–14 µm. Hence, additional optimization of the structure height and surface chemistry [23,40,46] may yield better control and enhancement of bacterial colonization on the surfaces. 

## 5. Conclusions

In this study, we established a process for the fabrication of honeycomb patterns with different periodicities and different morphologies in silicon surfaces. Our results suggest that such structures may promote biofilm formation, but the effect of the surface morphology parameters was inconclusive. Our results are encouraging, as the applied fabrication methods allow a wide range of patterns and aspect ratios and are scalable with respect to surface area. A huge parameter space, including possible different choices of materials, awaits investigation. Further improvements of the proposed method and further optimization of surface structures and surface chemistries may hold promise for significantly enhanced bacterial biofilm growth. In addition, it is also necessary to understand how those patterned surfaces can be effectively integrated into industrial applications for the promotion of beneficial bacterial biofilms, before scaling the fabrication process to large areas.

## Data Availability

The data presented in this study are available on request from the corresponding author.

## Supplementary Materials

The following are available online at https://www.mdpi.com/article/10.3390/mi12080926/s1, Figure S1: Fabrication steps for acquiring microscope slide; Figure S2: Image of the experimental batch setup; Figure S3: Composite graph with the mean measured sizes of the different IDs of honeycomb pillars; Figure S4: Composite graph with the measured sizes and dimensions of the different Reversed IDs of honeycomb pits; Figure S5: Composite graph illustrating the average bacterial biofilm biomass (µm^3^ µm^−2^) (bacterial volume/surface) of *Phaeobacter inhibens* biofilm, grown at 700 rpm after 96 hours. Table S1: Bosch process PrD-4 used for dry etching to a target depth of 10 μm; Table S2: Plasma ashing parameters to remove AZ 5214E photoresist; Table S3: Honeycomb pillars nominal dimensions used for the design. Nine different patterned surfaces with arrays of pillars were fabricated. Each different ID (H) was fabricated based on the nominal trench widths, side lengths and heights that are shown; Table S4: Honeycomb pits nominal dimensions used for the design. Nine different patterned surfaces with arrays of pits were fabricated. Each different Reversed ID (R) was fabricated based on the theoretical wall widths, side lengths and heights that are shown.
