# Supplementary material for: Fabrication of Microstructured Surface Topologies for the Promotion of Marine Bacteria Biofilm
Source: Micromachines (Basel). 2021 Aug 3;12(8):926. doi: 10.3390/mi12080926 (PMC8401315; doi:10.3390/mi12080926)
Supplement: Supplementary file 1 [file micromachines-12-00926-s001.zip › micromachines-1318328-supplementary.pdf]

micromachines

Supplementary Materials

Fabrication of Microstructured Surface Topologies for the Promotion of Marine Bacteria Biofilm

Ariadni Droumpali, Jörg Hübner, Lone Gram and Rafael Taboryski

Abstract: Several marine bacteria of the *Roseobacter* group can inhibit other microorganisms and are especially antagonistic when growing in biofilms. This aptitude to naturally compete with other bacteria can reduce the need for antibiotics in large-scale aquaculture units, provided that their culture can be promoted and controlled. Micropatterned surfaces may facilitate and promote the biofilm formation of species from the *Roseobacter* group, due to the increased contact between the cells and the surface material. Our research goal is to fabricate biofilm-optimal micropatterned surfaces and investigate the relevant length scales for surface topographies that can promote the growth and biofilm formation of the *Roseobacter* group of bacteria. In a preliminary study, silicon surfaces comprising arrays of pillars and pits with different periodicities, diameters, and depths were produced by UV lithography and deep reactive ion etching (DRIE) on polished silicon wafers. The resulting surface microscale topologies were characterized via optical profilometry and scanning electron microscopy (SEM). Screening of the bacterial biofilm on the patterned surfaces was performed using green fluorescent staining (SYBR green I) and confocal laser scanning microscopy (CLSM). Our results indicate that there is a correlation between the surface morphology and the spatial organization of the bacterial biofilm.

Keywords: structured surfaces, silicon surfaces, microfabrication, bacterial biofilm, microbial adhesion.

Citation: Droumpali, A.; Hübner, J.; Gram, L.; Taboryski, R. Fabrication of Microstructured Surface Topologies for the Promotion of Marine Bacteria Biofilm. *Micromachines* 2021, *12*, 926. https://doi.org/10.3390/mi12080926

Academic Editor: Ion Stiharu

Received: 13 July 2021
Accepted: 29 July 2021
Published: 3 August 2021

Publisher's Note: MDPI stays neutral with regard to jurisdictional claims in published maps and institutional affiliations.

Copyright: © 2021 by the authors. Submitted for possible open access publication under the terms and conditions of the Creative Commons Attribution (CC BY) license (https://creativecommons.org/licenses/by/4.0/).

Supplementary Materials

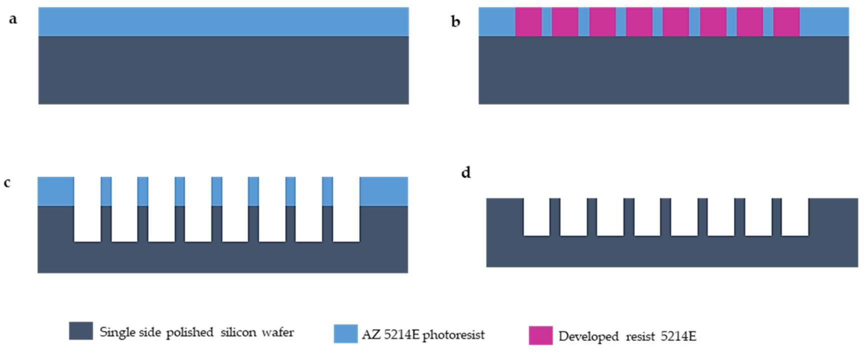

Figure S1. Fabrication steps for acquiring microscope slides.
Silicon master: Steps (**a**)-(**d**) are: (**a**) UV deposition of photoresist AZ5214E and UV exposure with MLA2; (**b**) Resist development; (**c**) Dry etching of silicon by a Bosch process; and (**d**) Plasma ashing of the resist. [1.]

Table S1: Bosch process PrD-4 used for dry etching to a target depth of 10 μm.

Main etch (D->E)	Etch	Deposition
**Gas flow (sccm)**	$SF_6$275 O_2 5	C_4F_8 150
**Cycle time (secs)**	2.4	2.0
**Pressure (mtorr)**	26	20
**Coil power (W)**	2500	200

**Platen power (W)**	2.5	5
**Cycles**	110 (process time 08:04)	
**Common**	Temperature 0 degs, HBC 10 torr, Long funnel, with baffle & 100 mm spacers	

Table S2: Plasma ashing parameters to remove AZ 5214E photoresist.

**Plasma ashing parameters**	
**Duration (min)**	30
**Pressure (mbar)**	1.25
**Power (W)**	1000
**O_2 flow (ml/min)**	400
**N_2 flow (ml/min)**	70

Table S3: Nominal dimensions of the honeycomb pillars used for the designs. Nine different patterned surfaces with arrays of pillars were fabricated. Each different ID (H) was fabricated based on the nominal trench width, side length, and height shown.

**Sample ID**	**Nominal trench width d (μm)**	**Nominal side length a (μm)**	**Nominal height (μm)**
**H 1**	1	2.5	10
**H 2**	1	5	10
**H 3**	1	10	10
**H 4**	2.5	2.5	10
**H 5**	2.5	5	10
**H 6**	2.5	10	10
**H 7**	5	2.5	10
**H 8**	5	5	10
**H 9**	5	10	10

Table S4: Nominal dimensions of the honeycomb pits used for the designs. Nine different patterned surfaces with arrays of pits were fabricated. Each different Reversed ID (R) was fabricated based on the theoretical wall width, side length, and height shown.

**Sample ID**	**Nominal wall width d (μm)**	**Nominal side length a (μm)**	**Nominal height (μm)**
**R 1**	1	2.5	10
**R 2**	1	5	10
**R 3**	1	10	10
**R 4**	2.5	2.5	10
**R 5**	2.5	5	10
**R 6**	2.5	10	10
**R 7**	5	2.5	10
**R 8**	5	5	10
**R 9**	5	10	10

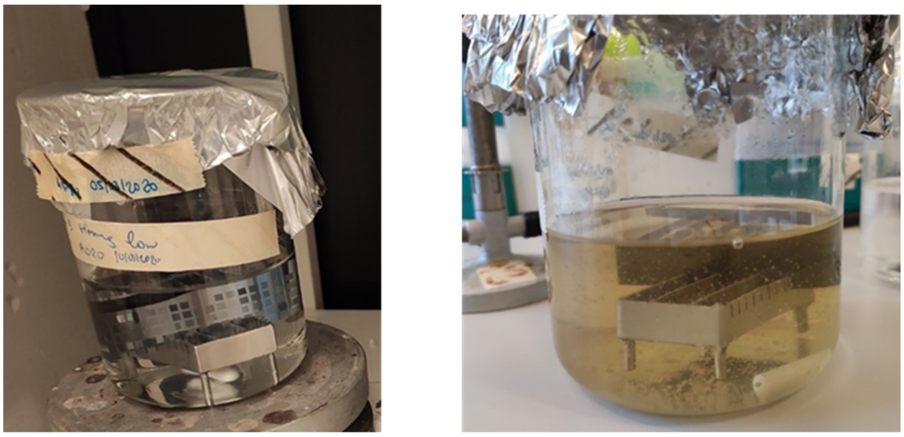

Figure S1: Images of the experimental batch setup consisting of a beaker, a metal rack, the silicon microscope slides on the rack, and the growth media: left, before inoculation; right, after inoculation. [1]

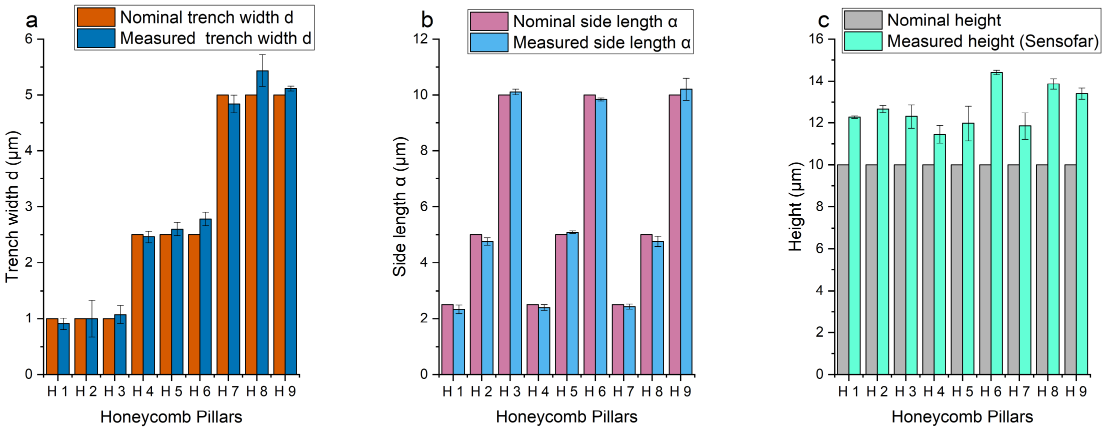

Figure S2: Composite graph with the mean measured sizes of the different honeycomb pillars (labeled by their IDs) compared with their nominal values in terms of (**a**) Trench width d; (**b**) Side length a; and (**c**) Height. Error bars represent standard deviations of the measured values.

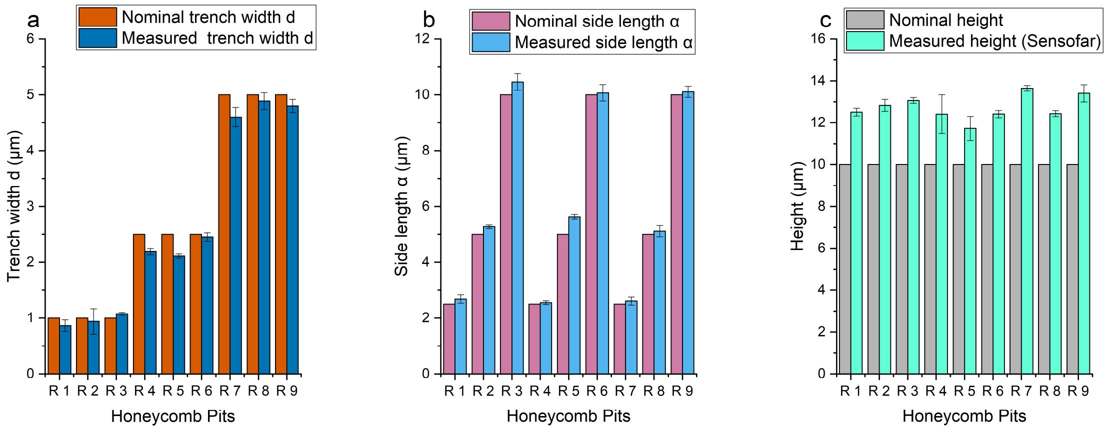

Figure S3: Composite graph with the measured sizes and dimensions of the different honeycomb pits (labeled by their Reversed IDs) compared with their nominal values in terms of (**a**) Wall width d; (**b**) Side length a; and (**c**) Height. Error bars represent standard deviations of the measured values.

The shown values are mean values from three replicates, and the error bars represent the standard deviations of those values. Figure S2a shows the mean measured trench widths of the different patterned honeycomb pillars from SEM measurements, in comparison with their theoretical values. Figure S3a expresses the mean measured wall widths of the different patterned honeycomb pits, measured by SEM, in comparison with their theoretical values. The graph in Figure S2b illustrates the mean measured side lengths a of the different patterned honeycomb pillars, measured by SEM, in comparison with their theoretical values, while the graph in Figure S3a illustrates the corresponding values for the different R specimens. In Figure S2c, the mean measured heights of the different patterned honeycomb pillars, measured by Sensofar, in comparison with their theoretical values are illustrated. In Figure S3c, the corresponding measured heights of all the patterned honeycomb pits (Rs) are presented.

In both figures, we can observe that the measured values for width d and side length a do not fluctuate from the theoretical ones. However, the measured heights on both pillars and pits are higher than the nominal values. This result is due to the D-RIE process, where etching of surfaces happens via a Bosch process and depends on so-called Aspect-Ratio-Dependent Etching or ARDE [2].

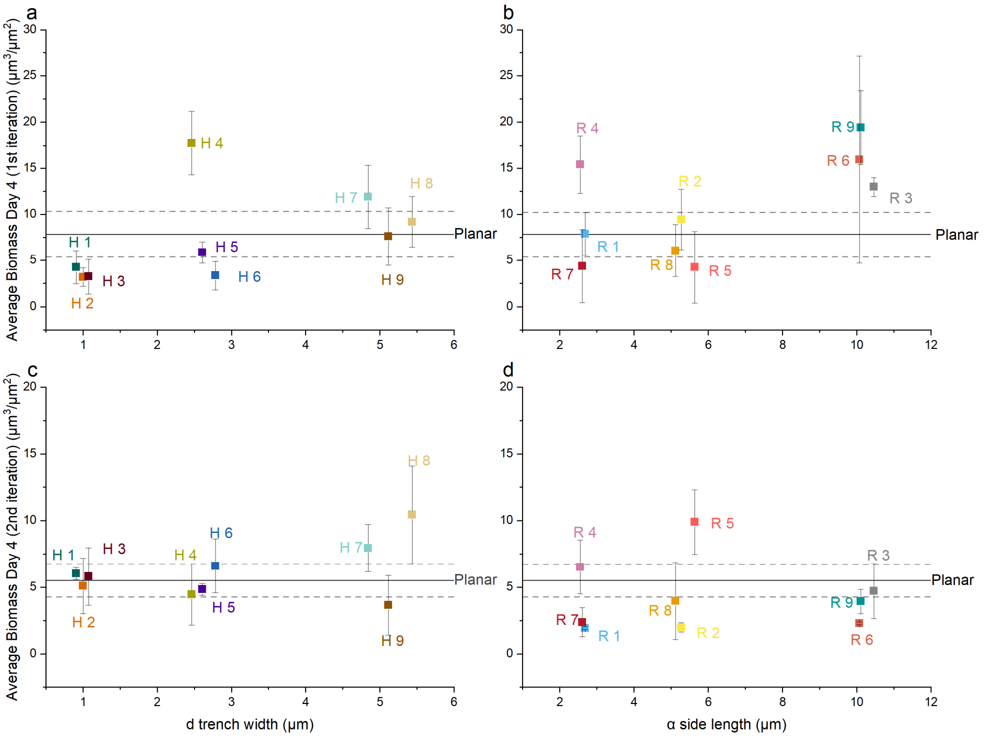

Figure S4: Composite graph illustrating the average bacterial biofilm biomass ($\mu m^3\ \mu m^{-2}$) (bacterial volume/surface) of *Phaeobacter Inhibens* biofilms, grown at 700 rpm for 96 hours, in comparison with the trench width d for pillars and side length a for pits; (**a**) Average biomass of bacterial biofilms grown on honeycomb pillars with different periodicities (1st iteration); (**b**) Average biomass of bacterial biofilms grown on honeycomb pits with different periodicities (1st iteration); (**c**) Average biomass of bacterial biofilms grown on honeycomb pillars with different periodicities (2nd iteration); and (**d**) Average biomass of bacterial biofilms grown on honeycomb pits with different periodicities (2nd iteration). Error bars represent standard deviations of the measured values from three technical replicates.

Bacterial attachment experiments were conducted on the patterned surfaces with increased shear stress (by increasing rpm) for 48, 96, and 192 hours. Our hypothesis was that by increasing the shear stress, we could have a better overview on which surfaces the bacteria would attach to, as in natural environments there is always a stream, which can be correlated with increasing the rpm in a closed system.

In Figure S4**Error! Reference source not found.**, the average bacterial biofilm biomass (μm³/μm²) of *Phaeobacter Inhibens* biofilm grown at 700 rpm after 96 hours is correlated with the different length scale parameters of the patterned surfaces. Figure S4a illustrates the average biomass of bacterial biofilms grown on honeycomb pillars, and Figure S4b shows the average biomass of bacterial biofilms grown on honeycomb pits with different periodicities.

The results indicate that the honeycomb pillars H4 and H7 have higher biomass than the planar surface (Figure S4a). This result can be associated with the lower side length on those patterns (2.5 μm) and higher trench width d between the pillars (2.5 μm or 5 μm, respectively). In Figure S4b, the results demonstrate that bacterial biofilm has a higher biomass on honeycomb pit surfaces with higher side length a, compared to the planar surface. Figure S4c and Figure S4d illustrate the average biomass of bacterial biofilms grown on honeycomb pillars and pits, respectively, during the second iteration (August 2020). In Figure S4c the patterned surface H8 with honeycomb pillars, trench width d of 5 μm, and side length a of 5 μm has the highest biomass growth. In Figure S4d, the patterned surface R5, with honeycomb pits, wall width d of 2.5 μm, and side length a of 5 μm, has the highest biomass growth compared to the planar surface. In both Figure S4c and Figure S4d, the results demonstrate that the number of biomasses on a planar surface is similar to that on patterned surfaces. Although the method was identical for the first and second iterations, the second iteration provided results that were not substantially different across the different patterns.

References

1. Droumpali, A.; Hübner, J.; Gram, L.; Taboryski, R. Fabrication of Micro-Structured Surface Topologies for the Promotion of Marine Bacteria Biofilm. In Proceedings of the Micromachines 2021—1st International Conference on Micromachines and Applications (ICMA2021, online conference, date: 15–30 April, 2021); MDPI: Basel, Switzerland, 16 April 2021; p. 9579.
2. Li, D. Aspect Ratio Dependent Etching. In *Encyclopedia of Microfluidics and Nanofluidics*; Springer: Boston, MA, USA, 2008; pp. 56–56. doi: 10.1007/978-0-387-48998-8
